# A 2 °C difference affecting the spatiotemporal distribution of small demersal fish assemblages in shallow tropical and subtropical waters of Western Taiwan

**DOI:** 10.1038/s41598-023-47300-8

**Published:** 2023-11-17

**Authors:** Kuo-Shu Chen, Chiee-Young Chen, Yi Chang, Hsu-Sen Chen, Meng-Hsien Chen

**Affiliations:** 1 Marine Ecology and Conservation Research Center, National Academy of Marine Research, Kaohsiung, 80661 Taiwan; 2 Department of Oceanography (Marine Biology Group), National Sun Yat-sen University, Kaohsiung, 80424 Taiwan; 3 Department of Marine Environmental Engineering, National Kaohsiung University of Science and Technology (Nanzih Campus), Kaohsiung, 81157 Taiwan; 4 Graduate Institute of Marine Affairs, National Sun Yat-sen University, Kaohsiung, 80424 Taiwan; 5 R/V New Ocean Researcher 3, Marine Instrument Center, College of Marine Sciences, National Sun Yat-sen University, Kaohsiung, 80424 Taiwan; 6 Department of Aquaculture, National Pingtung University of Science and Technology, Pingtung, 912301 Taiwan; 7 Institute of Marine Ecology and Conservation, National Sun Yat-sen University, Kaohsiung, 80424 Taiwan; 8 Water Resources Research Center, National Sun Yat-sen University, Kaohsiung, 80424 Taiwan; 9 Department of Biomedical Science and Environmental Biology, Kaohsiung Medical University, Kaohsiung, 80708 Taiwan

**Keywords:** Ecology, Ocean sciences

## Abstract

Two OR3 research vessel cruises were conducted at seven nearshore sites from north to south in the western coastal waters off Taiwan in May (late spring) and November (late autumn) 2019 in order to gain insights into the 2 °C difference in the tropical-subtropical fish fauna. Totally, 37 families, 72 genera, and 99 taxas were recorded for the 1809 fishes. Three fish assemblages, the North, South, and Fall-Zhuoshui River (ZRf) groups, were identified as eurythermal, stenothermal, and croaker communities. Their dominants, in rank order, were *Tarphops oligolepis*, *Liachirus melanospilosa*,* Ostorhinchus fasciatus*, and *Trachinocephalus myops* for the Northern eurythermal group, *Arnoglossus tenuis*,* Eubleekeria splendens*, and *Ostorhinchus pleuron* for the Southern stenothermal group, and *Johnius taiwanensis*, *Chrysochir aureus*, and *Pennahia macrocephalus* for the croaker-ZRf group. Their distribution was markedly correlated with the bottom water temperature, seafloor grain size, and concentration of suspended solids. The influence of a 2 °C difference on the tropical and subtropical demersal fish could indicate how rising temperatures due to climate change are shaping fish communities. The flatfish, *Liachirus melanospilos*, was distributed northward by 0.5^o^N and is suggested to be considered as an ecological indicator of the tropicalization of subtropical marine ecosystems in the future.

Water temperature is a key factor influencing the distribution of marine fishes^1–4^, resulting in different demersal fish assemblages being formed according to seasonality^4,5^. Geographical and habitat features are also important, for example location^6,7^, topographic features^2,8^, substrate type^2,4^, local hydrographic characteristics^5,9^, and biological factors such as estuarine seasonal migration^9^, and reproductive movement^9,10^. Fishing and other anthropogenic activities can also have substantial impacts on assemblages^11^.

According to the 2022 Intergovernmental Panel on Climate Change (IPCC) report, by the end of this century, the world sea water temperature will increase by 2 °C in comparison with the pre-industrial period^12^. The rising sea temperature has caused the tropicalization of fish assemblages in temperate areas^13^, and the range expansion of tropical reef fishes^14,15^. However, research on tropical-subtropical sandy bottom ecosystems has been limited^16^.

As a tropical and subtropical island, Taiwan’s climate, marine biodiversity, and seafood industry are strongly affected by the surrounding three major ocean currents, i.e., the warm Kuroshio Branch Water (KBW), the warm South China Sea Surface Water (SCSSW), and the cold China Coastal Water (CCW). These currents deliver warm/cold waters, thus influencing the surrounding sea water temperature, current direction, and sediment transport and deposition off the coast of the island^17–20^. Its largest river, the Zhuoshui (Choshui) River, is located in the central western region of the island and, over a period of millennia, its outflow has formed the Changyun Rise (CYR) off its river mouth in the Taiwan Strait. The rise forms an area with a water depth of about 40–50 m, which is 20–30 m shallower than other parts of the Taiwan Strait, creating a boundary which obstructs the flow of the warm KBW and SCSSW northward, and the cold CCW southward in winter, thus forming a north–south water temperature of at least *ca.* 2 °C and salinity of *ca.* 1 practical salinity units (psu) boundary in winter, but with less difference in summer^17,18^. Such a natural environmental boundary provides a great natural experimental location to test the null hypothesis that tropical-subtropical fish would not respond to a temperature difference of *ca.* 2 °C and form different fish assemblages accordingly. A previous study found the distribution of tropical reef fish species in southern Taiwan^21^. Zooplankton, e.g., copepods^22,23^, siphonophores^24,25^, euphausiids^26^, and shrimps^27^, have revealed different assemblages due to the large-scale influence of currents around Taiwan.

Furthermore, we also wanted to test the null hypothesis that no small demersal fish have moved northward, so as to gain insights into the adaptation of demersal fish in response to the decadal rising sea water temperature, which rose 3 °C from 1981 to 2012 in winter north of 23.5^o^N in Taiwan^28^, as well as to determine a possible ecological indicator to detect the ongoing warming marine ecosystem.

## Materials and methods

### Satellite image data

In order to understand the sea temperature difference and primary productivity along the latitudinal western coast of Taiwan prior to our survey, sea surface temperature (SST) and Chlorophyll concentration (Chl. *a*) images during the study period were archived from the Moderate Resolution Imaging Spectroradiometer aboard Aqua satellite (Aqua/MODIS) ocean color data set^29^ (https://coastwatch.pfeg.noaa.gov/). The SST and Chl. *a* data derived from Aqua/MODIS agreed well with in situ measurements around the study area, with root mean square errors of 0.75 °C^30^ and 0.1 mg/m^3^^31^, respectively. Both satellite-derived SST and Chl. *a* images from Aqua/MODIS have spatial resolution of 0.04 degrees (in latitude and longitude) and temporal resolution in daily products. Additionally, the geostrophic current field with a spatial resolution of 0.2 degrees and daily temporal resolution derived from the same data source were also archived. These satellite images were then integrated into 14-day composite maps before the day of our onboard field survey to illustrate fine features of SST variations and Chl. *a* concentrations associated with the current systems, enabling us to clarify the variability of fish assemblages relevant to physical environment changes in the Taiwan Strait. The array mathematics and image plotting of all satellite-derived datasets were processed using the software MATLAB R2022a of MathWorks, Inc. (https://www.mathworks.com/products/new_products/release2022a.html). Using Quantum GIS (QGIS, http://qgis.org), we conducted geo-mapping of satellite-based SST and Chl-*a* data.

### Study area

The study area is located on the nearshore coast of western Taiwan, in the tropical-subtropical climate zone (21° 54′–25° 18′ N, 120° 04′–121° 59′ E). This coastal area receives river runoff of 1900–2700 mm/yr from four major rivers, namely the Zhuoshui (Choshui) River, the Tsengwen River, the Dajia (Tachia) River, and the Kaoping (Gaoping) River, yielding annual sediment loads and deposition rates in descending order as follows: Zhuoshui River (63 × 10^6^t/yr, 20,000 t/km^2^/yr,), Kaoping River (36 × 10^6^t/yr, 11,000 t/km^2^/yr), Tsengwen River (31 × 10^6^ t/yr, 26,000 t/km^2^/yr), and Dajia (Tachia) River (3.6 × 10^6^ t/yr, 2900 t/km^2^/yr)^32^. The annual sediment deposition rates of the Zhuoshui and Tsengwen Rivers are both ranked no.1 in the world in the high mountain (> 3000 m) and 1000–3000 m mountain rivers categories, respectively^32^. These deposits form a soft bottom environment suitable for various tropical-subtropical marine demersal fishes.

**Figure 1:**
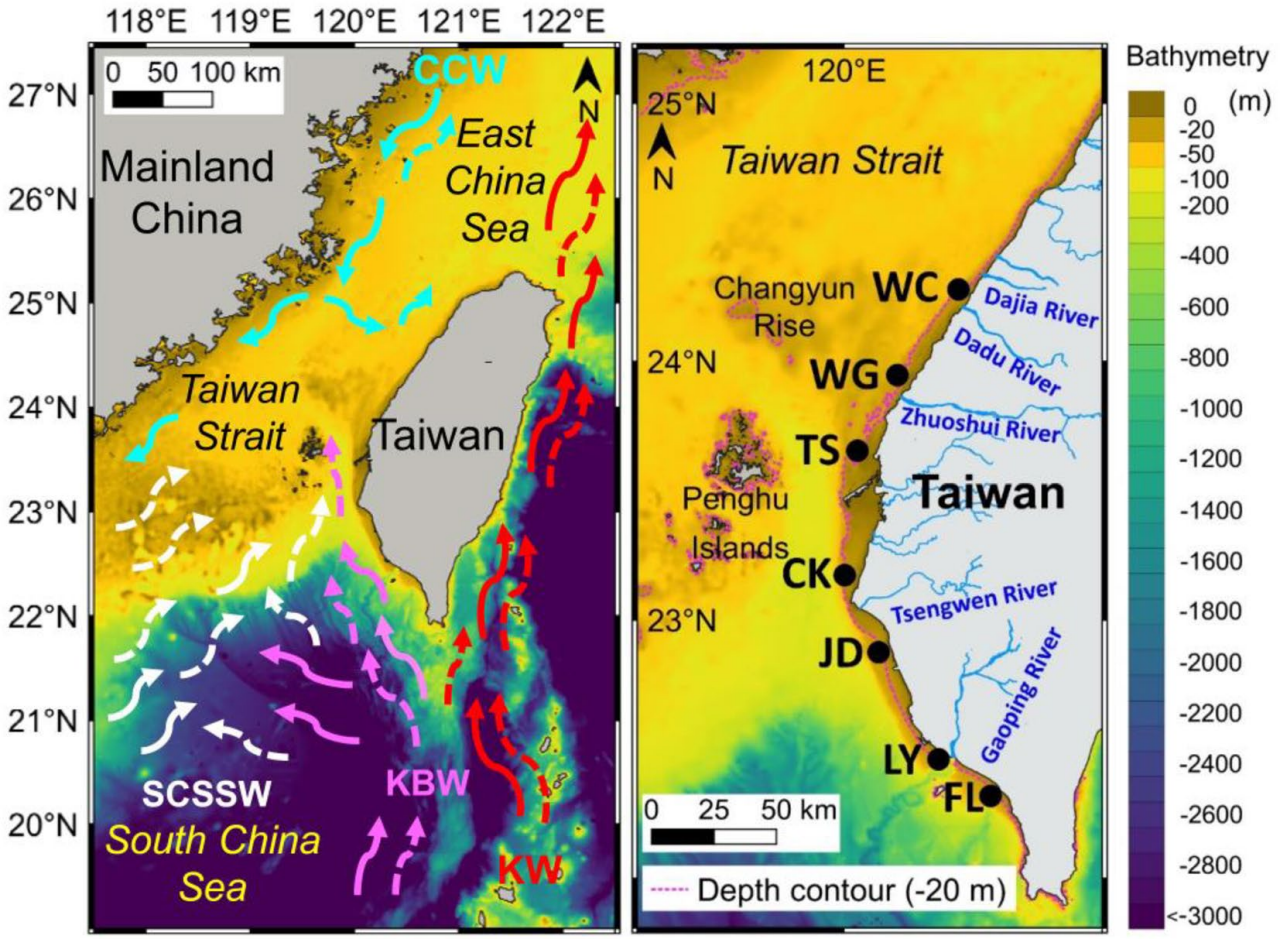
Map on the left shows the geographical location of Taiwan and the major seasonal surface water masses and their flowing direction (the dashed arrow for summer and the solid arrow for winter), i.e., KW = Kuroshio Water in red, KBW = Kuroshio Branch Water in pink, SCSSW = South China Sea Surface Water in white, and CCW = China Coastal Water in light blue around Taiwan. The map on the right shows the seven sampling sites (●), i.e. WC = Wuchi, WG = Wanggong, TS = Taisi, CK = Chiku, JD = Jiading, LY = Linyuan, and FL = Fangliao from north to south in the coastal waters off western Taiwan. This map was produced by QGIS 3.18 (QGIS.org, 2023).

In order to elucidate the impact of the 2 °C temperature difference on the small demersal fish assemblages, we carried out a north–south investigation in conjunction with Chen et al.^27^’s survey. Two seasonal cruises, on 25th to 27th May (late spring) and 16th to 18th November (late autumn), 2019 were conducted at seven sites along the western coastal waters off Taiwan. From the north to the south, the sites were Wuchi (Wuqi) (WC) (24° 15′ N), Wanggong (WG) (23° 59′ N), Taisi (Taixi) (TS) (23° 39′ N), Chiku (Qigu) (CK) (23° 09′ N), Jiading (Qieding) (JD) (22° 52′ N), Linyuan (LY) (22° 28′ N), and Fangliao (FL) (22° 18′ N) (Fig. 1). These sites were chosen according to their latitudinal position, topographic features, environmental conditions near estuaries and fishing ports, and the degree of influence of the seasonal large-scale currents that form a seasonal sea temperature gradient. The northernmost site, WC, is located off the estuary of Dadu River; it is strongly affected by the CCW in winter and forms a fishing ground near the category 1 fishing port of Wuchi in central western Taiwan; it is one of nine large Taiwanese fishing ports. The WG site is located on the northern bank of the Zhuoshui River where it receives the most abundant sediment loading from the largest river of Taiwan, and is near the category 2 fishing port of Wanggong. The TS site is situated south of the CYR, and is the northernmost point of the KBW in winter and near the category 2 fishing port of Taisi. The CK site is located at the northern end of the Penghu Channel, and is the westward turning point for the KBW as it flows toward the west of the Taiwan Strait; moreover, it is the southernmost point of the CCW’s normal reach in winter^17,18^. It is off the Chiku lagoon near the category 2 fishing ports of Jianjun and Chingshan. The JD, LY, and FL sites are situated along the southwestern coast of Taiwan, and are affected by the KBW all year round. The JD site is located off the estuary of the Erren River near the category 2 fishing port of Shinda. The LY site is located on the northern bank of the second largest river in Taiwan, the Kaoping River, and near the category 2 fishing port of Jungyun. The southernmost site of FL is located near the category 2 fishing port of Fangliao, which is an area that has historically never been affected by the CCW.

### Sampling method and measurement

The surveys were conducted by R/V *Ocean Researcher No.3* using a beam trawl (width 6 m, body length 8 m, body1 mesh 4.5 cm, and cod end mesh 2.5 cm) operating at a water depth of between 15 and 35 m, approximately 3 nautical miles off the coastline. At each site, two sampling locations were set at a water depth of *ca.*15 m and *ca.*25 m, respectively. Trawling, water sampling, and hydrological in situ measurements were performed. The trawling samples of shrimp have been published in Chen et al.^27^. In total, 27 trawling hauls were conducted (Appendix ). For each trawling haul, the beam trawl was deployed to touch the seafloor at the sampling site, with a towing speed of *ca*. 2 knots for a period of 30 min. The fish specimens caught by each trawl haul were immediately extracted from the catches, roughly classified by species and frozen at − 20 °C on board.

Subsequently, all fish specimens were transferred to the laboratory on land for taxonomic identification and morphological measurement (e.g., fish length (TL, mm) and body mass (BM, g)). The identification of fish specimens was conducted to the lowest level of taxonomic hierarchy possible based on the reference books of Shen et al.^33^ and Nakabo et al.^34^, and the fish databases of Froese and Pauly^35^ and Shao^36^.

Environmental conditions, including surface and bottom water temperature, salinity, and dissolved oxygen (DO) were recorded by an onboard Conductivity–Temperature–Depth profiler (CTD) and auxiliary sensors (Sea-Bird Electronics, inc., U.S.A). Near-bottom water samples at the trawling sites were collected with the rosette water sampler system (Sea-Bird Electronics, inc., U.S.A.). Concentrations of nutrients (including nitrate, nitrite, phosphate, silicate, and ammonia), suspended particles, and chlorophyll *a* in the near bottom water samples were analyzed according to the methods of Meng et al.^37^ Specifically, for measuring Chl-*a* concentration, each water sample was filtered through a 0.45 mm GF/F filter paper, and Chl-*a* was extracted from the filter after 24 h immersion in 90% acetone at 4 °C in the dark. Afterwards, Chl-*a* concentrations were measured with a spectrophotometer (Hitachi U-5100).

Additionally, sediment samples were collected with a Smith-McIntyre grab (Rigosha & Co. Ltd., Japan) before each beam trawling. Each grab was 33 cm in length, 33 cm in width, with a depth varying between 15 and 30 cm depending on the substrate characteristics. We took a sub-sample from the surface 10 cm of each grab, weighing approximately 300 g. These surface sediment samples were stored in a  − 20 °C freezer on board. After being brought back to the laboratory, all the sediment samples were sun-dried for several days, and large particles, such as shells, debris, and gravel were sieved out using a 1.0 mm mesh sieve before further analysis.

For the analysis of the sediment grain size, the Beckman-Counter Particle Coulter (model LS-13 320) was used. According to the Wentworth scale^38^, sediment grain can be categorized as coarse sand (500–1000 μm), medium sand (250–500 μm), fine sand (125–250 μm), very fine sand (62.5–125 μm), silt (3.9–62.5 μm), and clay (< 3.9 μm).

For the measurement of organic matter in the sediment, the loss on ignition method was applied. Two gram subsamples were weighed and placed in empty crucibles, then dried at 105 °C overnight to obtain the initial dry weight (W_0_, g). Then, they were heated at 550 °C for 4 h in a temperature-controlled furnace. The final sample was then cooled in a desiccator and weighed to gain the final dry weight (W_1_, g). The organic matter content (%) was calculated as W_0_ minus W_1_ divided by W_1_, then multiplied by 100.

All the hydrological measurements, such as bottom water temperature, salinity and pH, the concentrations of nitrite, nitrate, phosphate, silicate, ammonia, chlorophyll *a*, and suspended solids concentration in the water samples, as well as the benthic grain size and organic matter measurement were measured^27^.

### Data analysis

The species abundance data of each haul were standardized as “abundance/swept area” according to the method of Pacunski et al.^39^ to represent the number of individuals in square kilometers for each species (i.e., ind./10^4^ m^2^).

Using Primer V7 (PRIMER-E: Plymouth, UK)^40,41^, we conducted principal coordinate analysis (PCO), an unconstrained ordination approach, to distinguish the distribution patterns of fish abundance data among sites. We conducted PCO based on haul-specific species abundance data, using the Bray–Curtis similarity distance to construct a resemblance matrix. Prior to the construction of the resemblance matrix, the haul-specific abundance data were transformed using “square root,” in order to increase the weight of low abundance species to analyze all of the fish species. Additionally, to examine the effect of sampling sites, permutational multivariate analysis of variance (PERMANOVA) was used to test assemblage similarity data. Note that the seasonal effect was not tested because of an insufficient sample size. From the PCO results, we could identify the distribution patterns of the fish assemblages to confirm our north–south group hypothesis, and we tested statistical significance between groups using PERMANOVA. In addition, we used the canonical analysis of principal coordinates (CAP), a constrained ordination approach, to identify the assemblage patterns according to the grouping results of PCO and PERMANOVA. In the plot of the CAP results, the overlay vectors of fish species were produced based on Pearson correlations greater than 0.4 to represent simple linear correlations of individual variables with the CAP axes by Primer V7. The collinearity between the environmental variables was examined by Pearson correlation. Using CANOCO 5.1^42^, Canonical correspondence analysis (CCA), a constrained ordination approach, was performed to find the correlation of the abundance data of the 29 dominant fish species, representing 88.6% of the total catch, to the 8 environmental variables (bottom sea water temperature, bottom salinity, concentrations of chlorophyll *a* fluorescence (Chl.* a*) and suspended solids (SS), as well as the percentages of various grain sizes and organic matter), in order to identify the most important hydrographic characteristics.

## Results

### Satellite image data, in situ water temperature, and chlorophyll a

**Figure 2:**
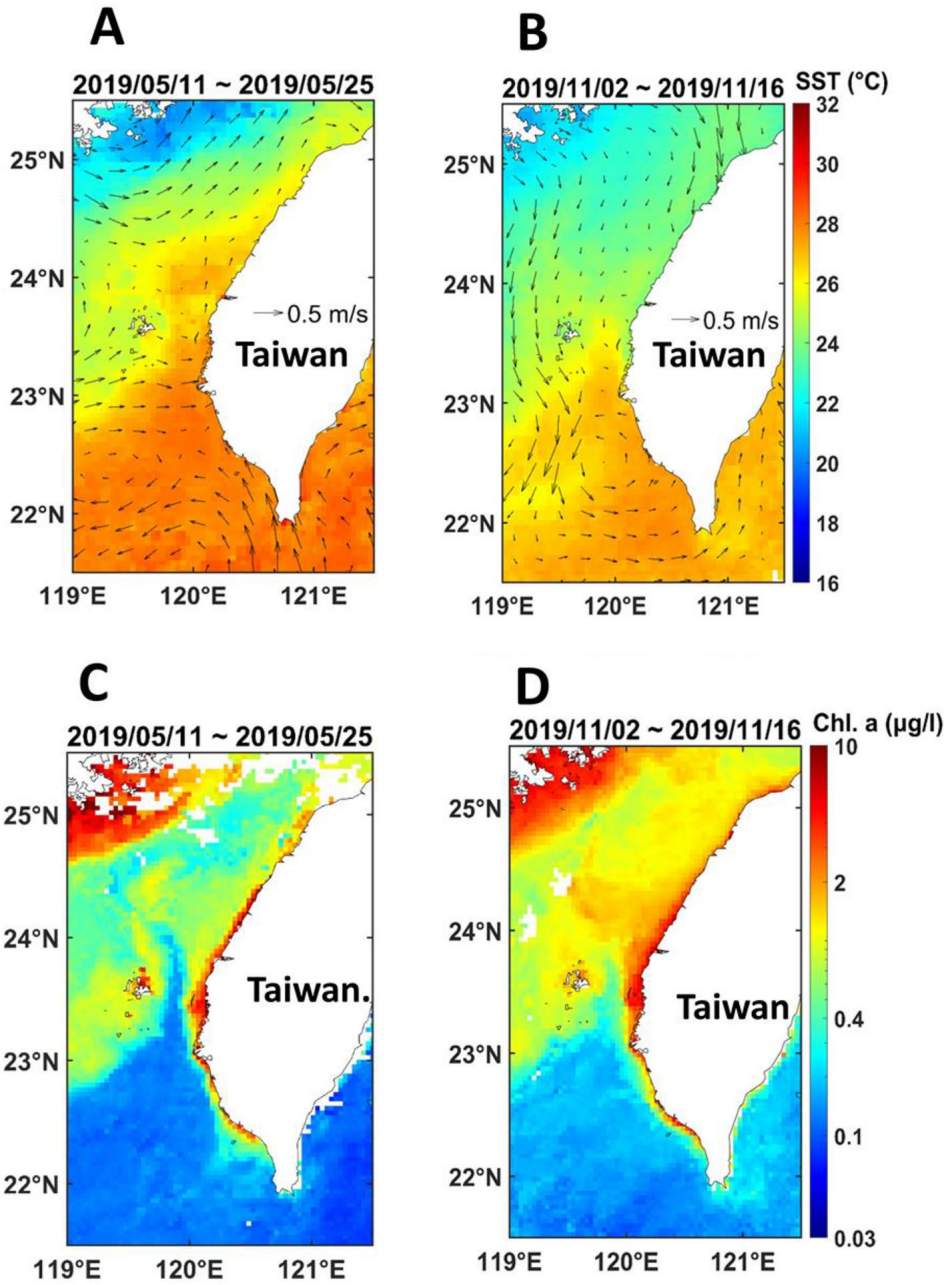
Images of satellite-based surface temperature (14 day-Aqua) in the upper panel (**A**) for 25th May and (**B**) for 16th November with geostrophic current (arrows), and chlorophyll-*a* concentration around Taiwan 14 days before the two surveys on (**C**) 25th May and (**D**) 16th November, 2019. The satellite-derived data source: https://coastwatch.pfeg.noaa.gov/.

From the satellite image, we can see the spatiotemporal difference in sea surface temperature. In May, the north–south temperature difference was between 26 and 29 °C in our survey area (Fig. 2A). The geostrophic current superimposed on the SST image showed that the strength of the CCW (< 22 °C) faded and turned northward because of the southwest monsoon which drove the KBW northward over the CYR, blocking the intrusion of the cold current CCW and forming an SST front difference (about 26 °C) along the north edge of the CYR. A clear 2 °C temperature difference (North: 27 °C vs. South: 29 °C) can be observed in May (Fig. 2A). Compared to in situ daytime measurements of the 7 latitudinal sites, mean bottom water temperature and the standard deviation were 27.53 ± 0.56 °C, ranging from 26.73 to 28.45 °C. The in situ mean bottom water temperatures in ascending order were 26.91 °C for WC, 26.79 °C for WG, 27.22 °C for TS, 27.63 °C for CK, 27.93 °C for JD, 27.85 °C for LY, and 28.39 °C for FL in May (Appendix 2).

Contrarily, the cold CCW intrudes into southern Taiwan from the north Taiwan Strait in November (Fig. 2B); particularly, part of the cold current intensifies and intrudes from the west Penghu Islands and combines with the warm KBW to contribute a cyclonic eddy in southern Taiwan. A north–south difference oceanic front was therefore found, with water temperatures varying from 24 to 28 °C with a significant split around the water at 23.20°N, north of the CK site. Therefore, a clear 2 °C temperature difference (North: 25 °C vs. South: 27 °C) can be seen in November (Fig. 2B). The in situ measurements of the 7 sites with mean bottom water temperature and the standard deviation were 25.73 ± 0.99 °C, ranging from 23.81 to 26.72 °C. The in situ mean bottom water temperatures in ascending order were 24.27 °C for WC, 24.69 °C for WG, 25.31 °C for TS, 26.11 °C for CK, 26.58 °C for JD, 26.54 °C for LY, and 26.62 °C for FL in November (Appendix 2).

The north to south in situ bottom temperature difference was *ca.*1.60 °C in May and *ca.* 2.25 °C in November. Furthermore, the temperature difference of each site between the two surveys showed a decreasing trend from north to south, i.e. *ca.* 2.64 °C for WC, *ca.* 2.10 °C for WG, *ca.* 1.91 °C for TS, *ca.* 1.52 °C for CK, *ca.* 1.35 °C for JD, *ca.* 1.31 °C for LY, and *ca.* 1.77 °C for FL. This result illustrates that there is a significant trend of the northernmost three sites showing a seasonal temperature difference of about 2–3 °C, whereas the southern sites show a smaller difference of *ca.* 1.5 °C. Notably, a marked sign indicating the cold water CCW (Temperature < 24 °C) southward flowing^18^ from the western Taiwan Strait into the coastal waters north of the CYR is that several low in situ bottom water temperatures of 23.81 °C and 24.73 °C at WC site, 24.22 °C at WG site, and 24.87 °C at TS site were recorded in November, coherent with the SST and current images.

From the satellite image, a significant northward warm KBW current in the southwestern coast of Taiwan can be seen in Fig. 2A; it forms a north–south temperature difference, raising the temperature to 26 °C at the WG site. In November, a strong southward cold CCW current coming from the northeast leads the cold water southbound to the TS site, reducing the water temperature to 24 °C. At the same time, the northward flowing warm KBW is restricted to the CK site, maintaining the water temperature at 27 °C (Fig. 2B).

Moreover, from the satellite image we can see that the Chl. *a* is always high along the western coast of Taiwan with a higher Chl. *a* at the CYR than in the other areas of the Taiwan Strait (Fig. 2C and D). Furthermore, a higher Chl. *a* was also observed in the northern part of the CYR in November in conjunction with the southbound CCW current (Fig. 2D). That agrees with our in situ measurement of Chl. *a* concentration on board, showing that there was an overall trend of lower concentrations in May (0.15 ± 0.15 μg/l) than in November (0.31 ± 0.30 μg/l) (Appendix 2).

Moreover, in Fall, the southbound CCW flows along the coast of China to Fujian; topographically its branch water makes an easterly turn and flows across the Taiwan Strait toward the western coast of Taiwan (Fig. 2B). This drives the elevated productivity (Fig. 2D) north of the CYR.

### Other hydrological conditions

The mean bottom salinity was measured as 34.02 ± 0.17 (33.8–34.2) and 34.02 ± 0.13 (33.67–34.26) at the seven sites in May and November, respectively, showing no significant spatiotemporal variation (Paired* t*-test, *p* > 0.05). On the contrary, the bottom water temperatures exhibited spatiotemporal differences between the two cruises (Paired *t*-test, *t* = 10.116, *df* = 6, *p* < 0.05). Additionally, pH values demonstrated temporal differences between the two cruises (Paired *t*-test, *t* = 20.467, *df* = 6, *p* < 0.05). The overall seasonal differences of SS concentrations were 7.87 ± 3.31 mg/l for May and 15.94 ± 9.87 mg/l for November (Paired* t*-test, *t* =  − 2.505, *df* = 6, *p* < 0.05). Notably, a surprising exceptionally high SS concentration found at the 15 m and 25 m measuring locations at the WG (34.67 and 14.00 mg/l, respectively) and TS (40.89 and 19.67 mg/l, respectively) sites in November was at least double all other measurements (4.78–17.56 mg/l) in the two surveys. As demonstrated in Appendix 2, various nutrient concentrations (in mg/l) showed no significant spatiotemporal differences between May and November i.e., NO_3_-N: 0.014 ± 0.008 vs. 0.022 ± 0.012 mg/l, NO_2_-N: 0.001 ± 0.001 vs. 0.003 ± 0.004 mg/L, PO_4_-P: < 0.002 vs. < 0.002 mg/l, SiO_2_-Si: 0.059 ± 0.040 vs. 0.078 ± 0.036 mg/l (Paired *t*-test, *p* > 0.05)), except for ammonia (NH_3_-N) concentration (NH_3_-N: 0.005 ± 0.004 vs. 0.014 ± 0.013 mg/l (Paired* t*-test, *t* =  − 2.542, *df* = 6, *p* < 0.05).

### Benthic grain size and organic matter

As shown in Appendix 2, the substrate composition was mainly sandy (63–1000 µm) with differences among sites rather than between seasons. In general, the sediment grain size had a boundary between the CK and JD sites. The sediment in the four northern sites was coarser (WC, WG, TS, and CK), with mainly fine sand, medium and coarse sand from 49.93 to 85.47% in May and 17.53–82.03% in November; on the other hand, in the three southern sites (JD, LY, and FL), the sediment was composed mainly of clay, silt, and very fine sand, with a range of 52.73–97.34% in May and 68.97–79.61% in November. The percentages of organic matter in the sediment for all seven sites are slightly lower in May than in November, i.e., 2.77 ± 0.81 (1.66 ~ 3.96%) vs. 3.05 ± 1.13 (1.72 ~ 5.63%). More specifically, a north–south site difference trend was found. That is, the four northern sites (2.33 ± 0.70; 1.66 ~ 4.72%) had lower percentages than the three southern sites (3.68 ± 0.73; 2.89 ~ 5.63%) in both May and November (*p* < 0.05, Mann–Whitney U test).

### Fish assemblages’ composition

In total, 37 families, 72 genera, and 99 taxa/species of benthic fish were recorded for the 1809 fish collection. A similar number of species was recorded in May (26 families, 51 genera, 65 taxa) and November (31 families, 49 genera, 63 taxa), but with a wide variety of fish compositions. Thirty-six species belonging to 19 families and 32 genera were recorded only in May, while there were 34 species belonging to 18 families and 24 genera that only occurred in November**.** The overall top five dominant fishes in terms of total abundance (ind./10^4^m^2^) in descending order were *Arnoglossus tenuis*, *Ostorhinchus fasciatus*, *Eubleekeria splendens*,* O. pleuron*, and *Tarphops oligolepis*, with a different spatial distribution of the top 5 dominant fish of each season (Table 1).

**Table 1:** List of the top 29 non-commercial fish (by total ranking and each cruise ranking) in abundance (ind./10^4^m^2^) collected at seven sites, *i.e.*, WC = Wuchi, WG = Wanggong, TS = Taisi, CK = Chiku, JD = Jiading, LY = Linyuan, and FL = Fangliao, along the western coastal waters off Taiwan during two research survey cruises (25th–27th May and 16th–18th November) in 2019. Note that a blank for fish abundance in the table indicates a zero value.

Total ranking	Fish code	Species or taxon	May, 2019								November, 2019							
			WC	WG	TS	CK	JD	LY	FL	Ranking	WC	WG	TS	CK	JD	LY	FL	Ranking
1	Ate	*Arnoglossus tenuis*		0.45			20.70	17.55	18.90	2					9.90	25.20	18.45	1
2	Ofa	*Ostorhinchus fasciatus*	8.55	2.25	3.60	1.80	6.30	2.70	11.70	4	7.20		0.45	18.00	3.15	3.60	4.95	2
3	Esp	*Eubleekeria splendens*					9.90	49.95		1					8.55	0.90		15
4	Opl	*Ostorhinchus pleuron*						7.65	44.10	3								27
5	Tol	*Tarphops oligolepis*	0.90			20.25				7	2.70			18.00				6
6	Egr	*Engyprosopon grandisquama*		0.45		0.45	9.00	5.40	11.70	5		0.45				0.45	12.60	11
7	Lme	*Liachirus melanospilos*		2.70		5.40	0.45			11		0.90		17.10			9.45	4
8	Pvo	*Pegasus volitans*					9.45		3.60	9						0.45	22.05	5
9	Jta	*Johnius taiwanensis*								23	0.45	18.90	16.20					3
10	Sma	*Suggrundus macracanthus*					5.40	0.45	14.40	8						1.35	9.45	13
11	Emu	*Engyprosopon multisquama*								23				6.75	12.60			7
12	Cau	*Chrysochir aureus*								23		3.15	15.75					8
13	Pma	*Pennahia macrocephalus*								23	1.35	4.05	13.50					9
14	Tmy	*Trachinocephalus myops*				2.25			0.90	17	10.35			4.05				10
15	Uja	*Upeneus japonicus*	2.70		18.00	0.45			1.35	6	1.80							25
16	Sae	*Sillago aeolus*								23	12.15							12
17	Ani	*Apogonichthyoides niger*			5.40	6.30				10	0.45			2.25				23
18	Ppa	*Pennahia pawak*		0.45						22	6.30	1.35	2.70					14
19	Ija	*Inegocia japonica*	0.90				5.85		0.45	13	2.70				0.45			22
20	Jtr	*Jaydia truncata*								23	5.40	2.25	0.45			0.45		16
21	Cpl	*Callionymus planus*		1.80			1.35			16				4.05	1.35			17
22	Pja	*Pseudogobius javanicus*					7.65			12								27
23	Cko	*Cynoglossus kopsii*				0.45	1.80			19	1.80	0.45		2.70				18
24	Gsp	Gobiid spp.					0.45	0.45	1.35	20	0.45			0.45		1.35	1.35	21
25	Emo	*Engyprosopon mozambiquensis*							4.95	15							0.45	26
26	Cbi	*Cynoglossus bilineatus*	0.45	1.80		0.45				18	1.35	0.45	0.45	0.45				24
27	Cin	*Cynoglossus interruptus*	1.80				3.60			14								27
28	Cpu	*Cynoglossus puncticeps*		0.90			0.45			21	3.60			0.45				20
29	Pse	*Polydactylus sextarius*								23	4.50		0.45					19
		Others (70 taxa)	2.70	3.15	9.90	4.50	12.60	4.95	13.50		21.15	6.75	1.35	2.25	0.90	2.25	8.55	
		Total number of species	10	14	9	17	27	16	27	66	36	16	12	17	9	13	16	72

### Spatiotemporal variation in the fish assemblages

Site difference trends in terms of species number were found in May and November, with a distinct northern/southern split in the distribution of the fish assemblages at the boundary between the CK and JD sites (Table 1).

**Figure 3:**
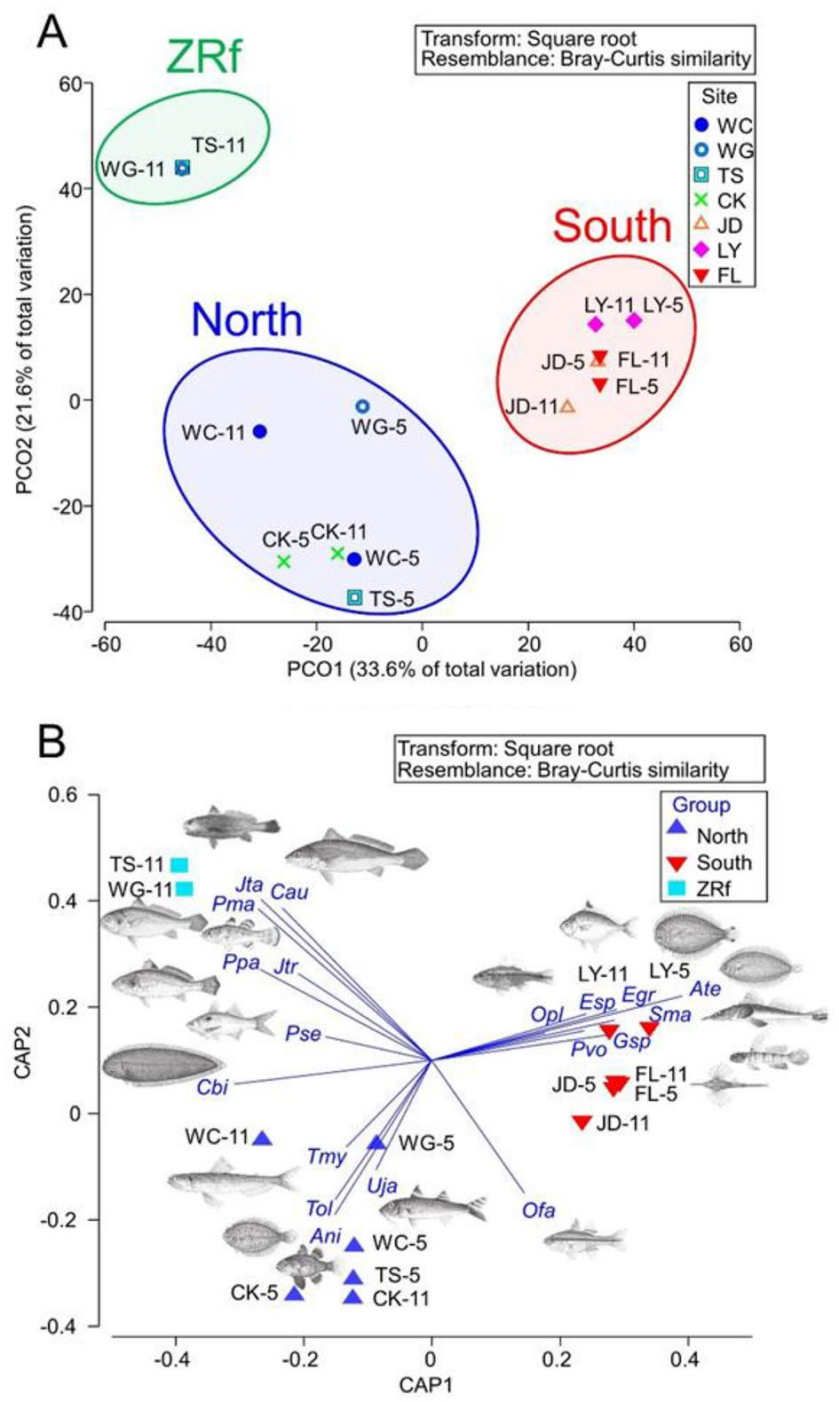
The first two axes from (**A**) the unconstrained ordination using principal coordinates analysis (PCO) of fish composition data and the abbreviations of the seven sites and the survey month are marked as XX-5, and XX-11 for May and November, respectively. The seven sampling sites, i.e. WC = Wuchi, WG = Wanggong, TS = Taisi, CK = Chiku, JD = Jiading, LY = Linyuan, and FL = Fangliao, in the coastal waters off western Taiwan, signify three hypothetical fish assemblages, the North, South and ZRf (Fall-Zhuoshui River) groups, and from (**B**) the constrained ordination using canonical analysis of principal coordinates (CAP) of species and sites with overlay vectors of species (Pearson correlation > 0.4), according to the hypothetical grouping of the North, South and ZRf groups, with the fish code shown in Table 1.

The top 29 abundant fish species among the total 99 fish taxa, composing 88.6% of the total catch in terms of relative abundance (87.8% and 89.5% in May and November, respectively) (Table 1) were selected to conduct the multivariate ordination analyses of PCO and CAP. The PERMANOVA test demonstrated a significant site effect of fish assemblages (*p* < 0.05; Table 2). Moreover, the PCO results rejected our null hypothesis, showing significant north–south fish assemblages, splitting at the CK and JD sites, except for the November samples collected at the WG and TS sites, which were classified as a small subgroup (Fig. 3A). With the three axes of PCO, they explain 67.3% of the total variance. Accordingly, we named the three groups of fish assemblages as the South, North, and ZRf (Fall-Zhuoshui River) assemblages (Fig. 3A). Furthermore, PERMANOVA pair-wise tests were performed to test the significance between any two groups, and also showed significance between the assemblage groups (*p* < 0.05; Table 3). Additionally, the CAP results revealed the spatiotemporal distribution patterns of the dominant species that were related to the assemblage groupings of the North, South, and ZRf groups, presenting different dominants (Fig. 3B).

**Table 2:** PERMANOVA table of results showing significant difference among sites for the fish assemblages at seven sites (see Fig. 1) in the shallow coastal waters off western Taiwan.

Source	*df*	SS	MS	Pseudo-*F*	*p* (perm)
Site	6	26,332	4388.7	2.3396	0.0053
Season (region)	7	13,131	1875.8	No test	
Total	13	39,463

**Table 3:** Results of permutational multivariate analysis of variance testing for differences based on the hypothetical grouping of fish assemblages in the coastal waters off western Taiwan.

Comparison	*t*	*p*-value
North, South	2.413	0.0018
North, ZRf	1.979	0.0383
South, ZRf	3.037	0.0359

### Correlation between environmental factors and fish assemblages

The correlation between the 8 environmental characteristics and the 29 dominant fishes of the South, North, and ZRf groups was examined by bi-plot CCA in order to test the null hypothesis that heterogeneity of hydrographic and sedimentary features would not affect the distribution of the small demersal fish fauna. The results of collinearity between the environmental variables are shown in Appendix 3. The bottom-water temperature was negatively correlated with the concentration of suspended solids (SS). Salinity was negatively correlated with the percentage of clay, silt, and very fine sand (CSVFS%), but positively correlated with the percentage of fine sand (FS%). The percentage of organic matter (OM%) was positively correlated with CSVFS%, but negatively correlated with the percentage of medium and coarse sand (MCS%). CSVFS% was negatively correlated with both FS% and MCS%.

**Figure 4:**
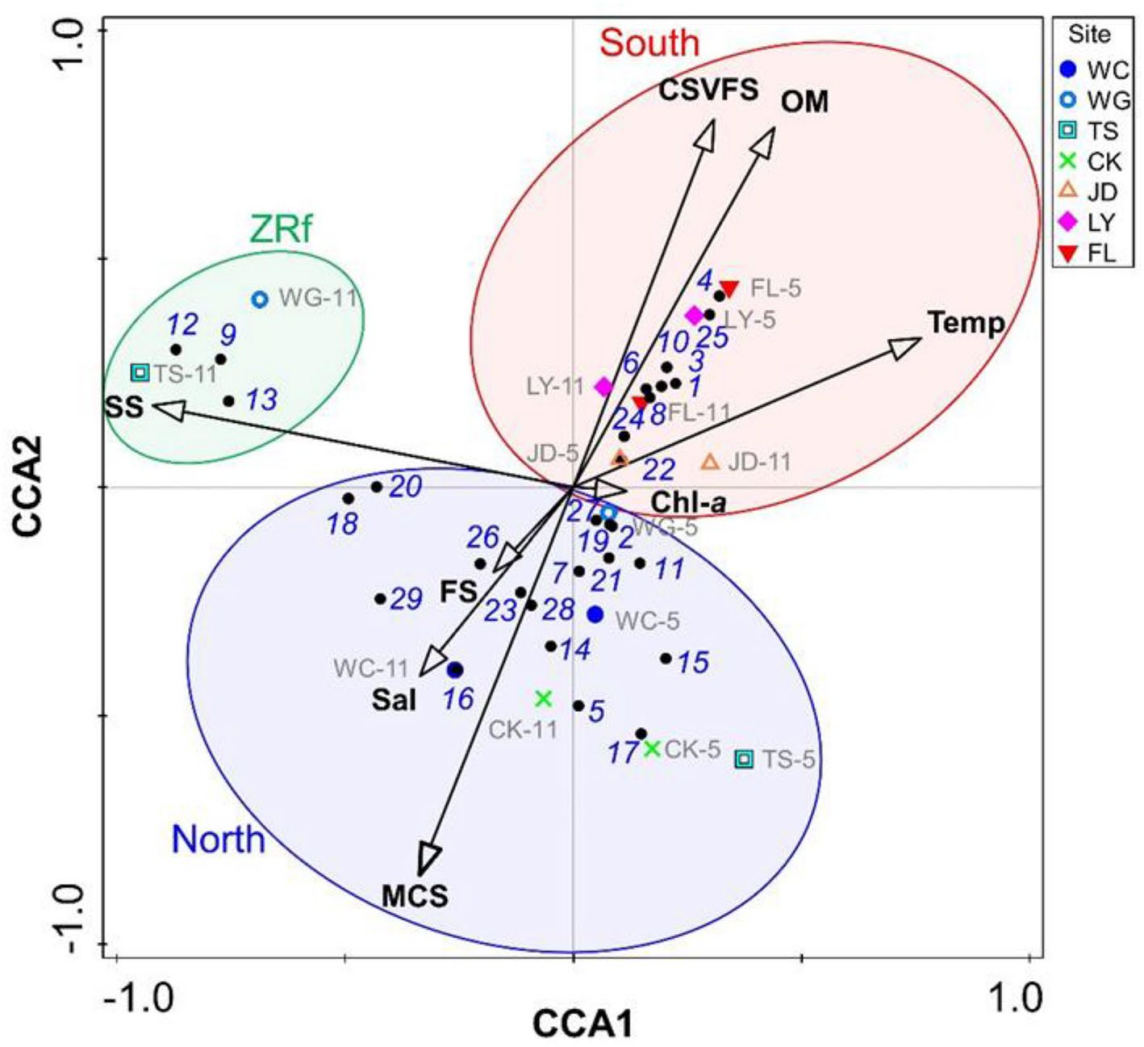
Bi-plot of the first two axes from the canonical correspondence analysis (CCA) of species, sites and environmental variables. Arrows from the origin demonstrate the various significant environmental variables. Black solid circles represent 29 dominant fish species marked by their total ranking list in Table 1. The abbreviations of the seven sites and the survey month are marked as XX-5, and XX-11 for May and November, respectively. The abbreviations of WC = Wuchi, WG = Wanggong, TS = Taisi, CK = Chiku, JD = Jiading, LY = Linyuan, and FL = Fangliao are for the seven sites, and Temp = seawater temperature, Sal = Salinity, SS = concentration of suspended solids, Chl-*a* = chlorophyll *a* fluorescence, CSVFS = percentage of clay, silt and very fine sand, FS = percentage of fine sand, and MCS = percentage of medium and coarse sand are for the environmental variables.

The CCA results rejected the hypothesis and showed that each group of fish was significantly affected by specific environmental factors (Table 4). The contrasting trends revealed CSVFS%, OM%, and bottom seawater temperature for the South group, MCS%, FS%, and bottom salinity for the North group, and SS for the ZRf group (Fig. 4, Table 4). Apart from these 8 hydrological and sediment factors, the remaining 10 environmental factors including various nutrient concentrations did not show any significant correlation to the abundance of the dominant fishes.

**Table 4:** Results of canonical correspondence analysis (CCA) for linking environmental variables to fish abundance for demersal fish assemblages off western Taiwan. Permutation test on the first axis: pseudo-*F* = 2.0, *p* = 0.0004.

CCA results	Axis 1	Axis 2	Axis 3	Axis 4
Eigenvalues	0.704	0.532	0.274	0.239
Explained variation (cumulative, %)	23.86	41.89	51.17	59.23
Pseudo-canonical correlation	0.97	0.98	0.98	0.97
Explained fitted variation (cumulative, %)	31.26	54.88	67.04	77.67

## Discussion

### 2 °C difference driven by warm/cold currents shaping the fish assemblages

Seasonal large-scale water currents create at least a 2 °C difference in temperature gradient, shaping the demersal fish assemblages by forming two geographically distinct fish assemblages, i.e., the North and South groups, plus one unique croaker subgroup (ZRf group) in the western coastal waters off Taiwan. The result rejects the null hypothesis that demersal fish assemblages would not respond to the north–south water temperature difference; instead they respond in the same way as shrimp^27^. The fish assemblages were divided geographically in the Chiku (*ca.* 23**° **09′ N) coastal waters near the upper western end of the Penghu Channel. Such a split in the fish assemblages was the integrated result of temperature, current speed, river outflow, and anthropogenic nutrients which has formed a eurythermal fish assemblage inhabiting the in situ bottom sea water temperature between 23.81 and 28.45 °C in the north, a warm stenothermal fish assemblage living at the bottom sea water temperature ranging from 26.58 to 28.45 °C in the south, and a cold stenothermal croaker assemblage aggregating at sea temperatures between 23.81 and 25.31 °C in the Zhoushui river estuary in fall. The Northern eurythermal group consists of eurythermal species, e.g. *T. oligolepis*, *O. fasciatus*, *L. melanospilos*, and *T. myops*, that can tolerate water temperatures lower than 24 °C and higher than 28 °C with a wider water temperature range. The Southern stenothermal group includes warm stenothermal fish species, e.g. *A. tenuis*, *E. splendens*, and *O. pleuron*, that prefer water temperatures higher than 24 °C all year round, with a narrower high temperature range. Those croakers only found in the Fall showed a narrower temperature range at *ca.* 25 °C.

The North group fishes were distributed from the tropical to the subtropical region with a wider range. The most dominant, *T. oligolepis*, has been recorded at the CK site and is the top dominant fish of the site located at Marine Existing Use Area 1 (MEAU1) of Taijiang National Park (TJNP), where the water temperature range is 20.9–30.3 ℃^43^. That agrees with the findings of Yamamoto et al.^15^ who reported that the fish were distributed as far north as from central and southern Japan at 34^o^N, with water temperatures ranging from 20.0 to 30.5 °C, to northern Kagoshima at 32^o^N^44^, and to Wakasa Bay, Japan at 34^o^N, where the surface sea water temperature varies from 7–8 °C in winter to 27–28 °C in summer^45^. The other two North-group dominants, *L. melanospilos* and *O. fasciatus*, were also abundant in MEAU1 of TJNP. They experience the same temperature range as *T. oligolepis* of TJNP^43^ as widely distributed tropical-subtropical fishes. *L. melanospilos* were found in a Mangrove creek (10° 26′ 13″ N, 122° 26′ 14″ E) in the Central Philippines with temperatures from 32.8 ± 0.9 °C to 33.1 ± 1.1 °C^46^ and in northern Kagoshima at 32^o^N^44^. *O. fasciatu*s were also found in the Mediterranean Sea in Syrian marine waters at 35^o^N^47^ and on the coast off West India with a sea water temperature range of 25.9–29.4 °C^48^.

The South group fishes are high temperature tropical organisms. For example, *E. splendens* are distributed in the tropical waters of the Blanakan coast, West Java, where the sea temperature ranges from 26.0 to 29.0℃^49^, and on the coast of West India with a sea water temperature range of 25.9–29.4 °C^48^. *O. pleuron* are also distributed in tropical waters, with a sea water temperature of 25.9–29.4 °C in West India^48^, whereas *A. tenuis* are distributed in tropical to subtropical waters, including northern Kagoshima at 32^o^N^44^ and even far north to Wakasa Bay, Japan at 34^o^N where the surface sea water temperature varies from 7–8 °C in winter to 27–28 °C in summer^45^.

### Effects of benthic grain size and concentration of suspended solids

Apart from the preferred bottom water temperature range for each of the North, South, and croaker-ZRf groups, benthic grain size and SS concentration are also factors which influence their distribution. The results reject the null hypothesis that heterogeneity of hydrographic and sedimentary features would not affect the distribution of the small demersal fish fauna; such results are the same as those for shrimp^27^. The North group inhabits an environment with lower organic matter, *ca.* 2%, coarse grain size with FS%, MCS%, and CS% of *ca.* 65%, and less turbulent water with an SS concentration range of 6.78–13.33 mg/l. This result is consistent with Chen et al.’s^43^ results regarding the demersal fish assemblages of TJNP. The South group fishes favored an environment with high organic matter, > 3%, a finer sediment grain size environment with CSVFS% & FS% of *ca.* 88%, and low turbidity waters with an SS concentration range of 5.56–12.72 mg/l. These records agree with Ikejima et al.^50^ who reported that *E. splendens* were caught in a mangrove creek in Trang province, Thailand, which was characterized by a muddy substrate. In the case of the croaker-ZRf group, they appear in environments with less organic matter, *ca.* 2%, finer sediment grain size CSVFS% & FS% of *ca.* 74%, and very turbid waters with an SS concentration range of 24.33–30.28 mg/l introduced by the Zhuoshui River plume^51^. These records echo Hayase and Haron’s^51^ report of various predominant Sciaenid in the Merbok and Sangga river estuary, Malaysia, where there is high turbidity of up to 15.0–106.3 NTU (*ca.* SS concentration = 50–365 mg/l). Acha et al.^10^ also found that whitemouth croaker, *Micropogonias furnieri*, spawned at the innermost Rio de la Plata, Argentina, with turbidity of up to 150 mg/l. Their appearance also echoes a typical living habit for croakers to aggregate in murky estuaries to feed, spawn, and over-winter^10,53^.

The most dominant ZRf croaker, *J. taiwanensis*, is distributed in the coastal waters on both sides of the Taiwan Strait from the Zhoushan Islands of Zhejian to Fujian, Guangdong, and Hong Kong on the southeastern coast of mainland China, and from Taoyuan to Kaohsiung in the western coastal waters of Taiwan^54,55^. They spawn from April to October in Fujian waters^55^, China. In our November survey, their total length range was from 33.3 to 85.9 mm (54.1 ± 8.3 mm, n = 79) which is less than their minimum mature size (in standard length) of 118 mm for males and 125 mm for females^55^, indicating that they were the young of the year juveniles and sub-adults. This may be a result of following the southbound CCW current across the Taiwan Strait and gathering off the Zhuoshui River estuary to feed in November.

The second dominant species of the ZRf group was *P. macrocephalus*. They are widely distributed in tropical and subtropical areas in the coastal waters of Beibu Gulf, south of Hainan Island, China at 17–18^o^N^56^ and Ohnuki Beach, Tokyo Bay, Japan at 35^o^N^57^. The group of *P. microcephalus* composed two cohorts in our November survey. Their large size cohort with a total length of 112.2–180.6 mm (127.9 ± 12.6 mm, n = 34) were at the age of *ca*. 7–18 months^58^ and would reach their mature size of *ca.* 187–201 mm (18–22 month old)^58,59^ for females, and of *ca.*147–150 mm (11–13 months old)^58,59^ for males in the following year^59^. In comparison, their small size cohort of 57.2–65.6 mm (61.9 ± 2.9 mm, n = 8) were about 1.5–2.5-months-old^58^. Therefore, we speculate that the Zhuoshui River estuary is a feeding ground for their juveniles and pre-spawning adults in November. They would then reproduce their offspring from May to October, with a peak from July to September in the coastal waters off southwestern Taiwan^59^.

The third dominant species of the ZRf group was *C. aureus*, with a total length range of 5.1–94.4 mm (63.7 ± 14.3 mm, n = 42). They are mainly distributed in tropical waters, i.e., caught at Pahang estuary, Malaysia, where the water temperature range is 26.7–30.6 °C^60^. Their 50% sexual mature total length is 210 mm for males and 263 mm for females, which spawn from June to December in the southwestern waters of Taiwan^61^. Therefore, they are all young of the year juveniles and sub-adults in our November survey at the WC and TS sites. The size composition of the three dominants of the ZRf group imply that the Zhuoshui River estuary is a very important site for the croakers to feed and nurse their next or next two years’ brood stock. They specifically choose to feed and grow in this river estuary rather than in any of the others along the coast which fall within their suitable temperature range, as it has the highest levels of SS concentration due to the high anthropogenic input of nutrients in the Zhuoshui estuary through the rainy season from June to September before late fall.

### Northward movement of tropical fishes

*Liachirus melanospilos*^62^, the second dominant species of the North group, revealed their northward distribution. The null hypothesis that there is no fish movement northward for the tropical small demersal fish is rejected. *L. melanopilos* used to co-occur with *Parapenaeopsis hardwickii* and were abundant at JD in a late 1990s study^63^. They have expanded their distribution from the JD to the WG site, showing a trend of north range expansion of *ca.* 0.5^o^N with the most abundant at the CK site, echoing a similar northward distribution as *Parapenaeopsis hardwickii* in the previous study^27^. This is further biological evidence in response to a *ca*. 3 °C temperature increase in winter on the western coast of Taiwan in 1981–2013^28^ causing the distribution change of tropical and subtropical benthos. It is suggested that they could be considered as an ecological indicator of rising sea temperatures for a tropical-subtropical marine ecosystem in the future.

## Conclusion

The large-scale currents driving a natural 2 °C sea temperature difference provide us with insights into how rising temperatures are shaping the tropical and subtropical fish assemblages, forming eurythermal and warm stenothermal fish assemblages. The dominants of the eurythermal fish group, i.e., *O. fasciatu*s, *T. oligolepis*, and *L. melanospilos*, were widely distributed from tropical to subtropical areas (< 35^o^N). The dominants of the warm stenothermal fish group, i.e., *A. tenuis*, *E. splendens*, and *O. pleuron*, were confined to the tropical area, living in the environment with water temperatures higher than 24 °C. The strong outfall of the Zhoushui river in summer forms a high murky productive environment suitable for various croakers, i.e., *J. taiwanensis*, *P. macrocephalus*, and *C. aureus*, to nurse and to feed in Fall, thus forming a Fall-croaker assemblage. Compared with historical data, we found that the eurythermal *L. melanospilos* has shown a northward habitat trend over the past 20 years. However, with limited data, our findings need to be further verified in the future.

### Supplementary Information

Supplementary Information.

## Data Availability

The datasets used and/or analyzed during the current study are available from the corresponding author on reasonable request.
