# Supplementary material for: A 2 °C difference affecting the spatiotemporal distribution of small demersal fish assemblages in shallow tropical and subtropical waters of Western Taiwan
Source: Sci Rep. 2023 Nov 17;13:20113. doi: 10.1038/s41598-023-47300-8 (PMC10656438; doi:10.1038/s41598-023-47300-8)
Supplement: Supplementary file 1 — Supplementary Information. [file 41598_2023_47300_MOESM1_ESM.docx]

Appendix 1. A list of fish taxa and their individuals based on the grouping result obtained by multivariate ordination in this study (ZRf: Zoushui River fish group).

| Family | Species or taxon | Group | | | |
| --- | --- | --- | --- | --- | --- |
|  |  | North  (11 hauls) | South  (12 hauls) | ZRf  (4 hauls) | Total  (27 hauls) |
| Platyrhinidae | *Platyrhina tangi* | 0 | 0 | 3 | 3 |
| Dasyatidae | *Dasyatis akajei* | 1 | 0 | 0 | 1 |
| Dasyatidae | *Dasyatis bennettii* | 1 | 0 | 0 | 1 |
| Dasyatidae | *Dasyatis zugei* | 1 | 0 | 1 | 2 |
| Pristigasteridae | *Ilisha melastoma* | 7 | 0 | 0 | 7 |
| Engraulidae | *Setipinna tenuifilis* | 0 | 0 | 1 | 1 |
| Engraulidae | *Stolephorus insularis* | 1 | 0 | 0 | 1 |
| Plotosidae | *Plotosus lineatus* | 3 | 0 | 2 | 5 |
| Synodontidae | *Saurida wanieso* | 0 | 2 | 0 | 2 |
| Synodontidae | *Trachinocephalus myops* | 37 | 2 | 0 | 39 |
| Myctophidae | *Benthosema pterotum* | 7 | 0 | 0 | 7 |
| Bregmacerotidae | Bregmacerotid sp. | 0 | 1 | 0 | 1 |
| Antennariidae | *Antennarius striatus* | 0 | 1 | 0 | 1 |
| Pegasidae | *Pegasus volitans* | 0 | 79 | 0 | 79 |
| Solenostomidae | *Solenostomus cyanopterus* | 1 | 0 | 0 | 1 |
| Syngnathidae | *Hippocampus kuda* | 0 | 1 | 0 | 1 |
| Syngnathidae | *Trachyrhamphus serratus* | 1 | 0 | 0 | 1 |
| Dactylopteridae | *Dactyloptena peterseni* | 0 | 1 | 0 | 1 |
| Scorpaenidae | *Apistus carinatus* | 1 | 0 | 0 | 1 |
| Scorpaenidae | *Inimicus japonicus* | 0 | 1 | 0 | 1 |
| Scorpaenidae | *Inimicus sinensis* | 0 | 2 | 0 | 2 |
| Scorpaenidae | Scorpaenid sp. | 0 | 1 | 0 | 1 |
| Platycephalidae | *Grammoplites scaber* | 7 | 0 | 1 | 8 |
| Platycephalidae | *Inegocia japonica* | 8 | 15 | 0 | 23 |
| Platycephalidae | *Rogadius asper* | 0 | 5 | 0 | 5 |
| Platycephalidae | *Sorsogona tuberculata* | 2 | 3 | 0 | 5 |
| Platycephalidae | *Suggrundus macracanthus* | 0 | 69 | 0 | 69 |
| Platycephalidae | *Suggrundus meerdervoortii* | 6 | 0 | 0 | 6 |
| Serranidae | *Epinephelus awoara* | 1 | 0 | 0 | 1 |
| Apogonidae | *Apogonichthyoides niger* | 26 | 0 | 0 | 26 |
| Apogonidae | Apogonid sp. | 0 | 1 | 0 | 1 |
| Apogonidae | *Archamia bleekeri* | 0 | 2 | 0 | 2 |
| Apogonidae | *Jaydia lineatus* | 4 | 2 | 0 | 6 |
| Apogonidae | *Jaydia striata* | 0 | 2 | 0 | 2 |
| Apogonidae | *Jaydia truncata* | 12 | 1 | 6 | 19 |
| Apogonidae | *Ostorhinchus fasciatus* | 88 | 72 | 1 | 161 |
| Apogonidae | *Ostorhinchus pleuron* | 0 | 115 | 0 | 115 |
| Sillaginidae | *Sillago aeolus* | 27 | 0 | 0 | 27 |
| Sillaginidae | *Sillago asiatica* | 2 | 1 | 0 | 3 |
| Sillaginidae | *Sillago japonica* | 1 | 0 | 0 | 1 |
| Leiognathidae | *Eubleekeria splendens* | 0 | 154 | 0 | 154 |
| Leiognathidae | *Leiognathus berbis* | 3 | 3 | 0 | 6 |
| Leiognathidae | *Leiognathus ruconius* | 1 | 0 | 0 | 1 |
| Leiognathidae | *Photopectoralis aureus* | 0 | 6 | 0 | 6 |
| Leiognathidae | *Secutor indicius* | 0 | 3 | 0 | 3 |
| Gerreidae | *Gerres macracanthus* | 2 | 0 | 0 | 2 |
| Haemulidae | *Diagramma pictum* | 6 | 3 | 0 | 9 |
| Haemulidae | Haemulid sp. | 1 | 0 | 0 | 1 |
| Haemulidae | *Pomadasys argenteus* | 2 | 0 | 0 | 2 |
| Haemulidae | *Pomadasys kaakan* | 1 | 0 | 0 | 1 |

Appendix 1. Continued.

| Family | Species or taxon | | Group | | | |  |
| --- | --- | --- | --- | --- | --- | --- | --- |
|  |  |  | North  (11 hauls) | South  (12 hauls) | ZRf  (4 hauls) | Total  (27 hauls) |  |
| Nemipteridae | *Scolopsis affinis* | 0 | | 3 | 0 | 3 | |
| Polynemidae | *Polydactylus sextarius* | | 10 | 0 | 1 | 11 |  |
| Sciaenidae | *Chrysochir aureus* | | 0 | 0 | 42 | 42 |  |
| Sciaenidae | *Johnius borneensis* | | 0 | 0 | 1 | 1 |  |
| Sciaenidae | *Johnius taiwanensis* | | 1 | 0 | 78 | 79 |  |
| Sciaenidae | *Pennahia macrocephalus* | | 3 | 0 | 39 | 42 |  |
| Sciaenidae | *Pennahia pawak* | | 15 | 0 | 9 | 24 |  |
| Sciaenidae | *Protonibea diacanthus* | | 2 | 0 | 0 | 2 |  |
| Mullidae | *Upeneus japonicus* | | 31 | 3 | 0 | 34 |  |
| Mullidae | *Upeneus tragula* | | 2 | 2 | 0 | 4 |  |
| Drepaneidae | *Drepane punctata* | | 2 | 0 | 0 | 2 |  |
| Callionymidae | *Callionymus curvicornis* | | 0 | 10 | 0 | 10 |  |
| Callionymidae | *Callionymus doryssus* | | 0 | 9 | 0 | 9 |  |
| Callionymidae | *Callionymus planus* | | 13 | 6 | 0 | 19 |  |
| Callionymidae | *Dactylopus dactylopus* | | 0 | 1 | 0 | 1 |  |
| Gobiidae | Gobiid sp. | | 2 | 11 | 0 | 13 |  |
| Gobiidae | *Pseudogobius javanicus* | | 0 | 17 | 0 | 17 |  |
| Gobiidae | *Yongeichthys nebulosus* | | 0 | 1 | 0 | 1 |  |
| Stromateidae | *Pampus echinogaster* | | 0 | 0 | 8 | 8 |  |
| Paralichthyidae | *Pseudorhombus arsius* | | 1 | 0 | 0 | 1 |  |
| Paralichthyidae | *Pseudorhombus pentophthalmus* | | 0 | 1 | 0 | 1 |  |
| Paralichthyidae | *Pseudorhombus quinquocellatus* | | 1 | 4 | 0 | 5 |  |
| Paralichthyidae | *Tarphops oligolepis* | | 93 | 0 | 0 | 93 |  |
| Bothidae | *Arnoglossus tenuis* | | 1 | 246 | 0 | 247 |  |
| Bothidae | Bothid sp. | | 0 | 5 | 0 | 5 |  |
| Bothidae | *Bothus myriaster* | | 1 | 0 | 0 | 1 |  |
| Bothidae | *Crossorhombus azureus* | | 1 | 0 | 0 | 1 |  |
| Bothidae | *Crossorhombus valderostratus* | | 0 | 1 | 0 | 1 |  |
| Bothidae | *Engyprosopon grandisquama* | | 2 | 87 | 1 | 90 |  |
| Bothidae | *Engyprosopon maldivensis* | | 0 | 1 | 0 | 1 |  |
| Bothidae | *Engyprosopon mozambiquensis* | | 0 | 12 | 0 | 12 |  |
| Bothidae | *Engyprosopon multisquama* | | 15 | 28 | 0 | 43 |  |
| Soleidae | *Liachirus melanospilos* | | 56 | 22 | 2 | 80 |  |
| Soleidae | *Solea ovata* | | 6 | 0 | 0 | 6 |  |
| Cynoglossidae | *Cynoglossus arel* | | 1 | 4 | 0 | 5 |  |
| Cynoglossidae | *Cynoglossus bilineatus* | | 10 | 0 | 2 | 12 |  |
| Cynoglossidae | *Cynoglossus interruptus* | | 4 | 8 | 0 | 12 |  |
| Cynoglossidae | *Cynoglossus kopsii* | | 11 | 4 | 1 | 16 |  |
| Cynoglossidae | *Cynoglossus lida* | | 0 | 1 | 0 | 1 |  |
| Cynoglossidae | *Cynoglossus puncticeps* | | 11 | 1 | 0 | 12 |  |
| Cynoglossidae | *Cynoglossus* sp. | | 0 | 8 | 0 | 8 |  |
| Cynoglossidae | *Paraplagusia blochii* | | 1 | 0 | 0 | 1 |  |
| Triacanthidae | *Triacanthus biaculeatus* | | 1 | 0 | 0 | 1 |  |
| Monacanthidae | *Stephanolepis cirrhifer* | | 1 | 1 | 0 | 2 |  |
| Ostraciidae | *Tetrosomus gibbosus* | | 0 | 1 | 0 | 1 |  |
| Tetraodontidae | *Canthigaster rivulata* | | 0 | 1 | 0 | 1 |  |
| Tetraodontidae | *Lagocephalus sceleratus* | | 1 | 0 | 0 | 1 |  |
| Tetraodontidae | *Takifugu oblongus* | | 1 | 0 | 1 | 2 |  |
| Tetraodontidae | *Torquigener hypselogeneion* | | 1 | 0 | 0 | 1 |  |
| Total |  | | 562 | 1047 | 200 | 1809 |  |

Appendix 2. A list of hydrological parameters, nutrients (mg/l), Chlorophyll *a* (Chl-*a,* μg/l), and suspended solid (SS, mg/l) concentrations in near-bottom waters, and percentages of sediment grain size and organic matters measured at the seven sites, *i.e.*, WC, WG, TS, CK, JD, LY, and FL along the western coastal waters off Taiwan in the two research cruises during the periods of 25^th^–27^th^ May and 16^th^–18^th^ November, 2019. nd= non-detected limit (NO^2^-N: <0.001 mg/l; PO^4^-P: <0.002 mg l^−1^; NH^3^-N: <0.002 mg/l; Chl. *a*: <0.11 μg/l).

|  | May, 2019 | | | | | | |  | November, 2019 | | | | | | |  | Paired *t*-test |
| --- | --- | --- | --- | --- | --- | --- | --- | --- | --- | --- | --- | --- | --- | --- | --- | --- | --- |
|  | WC | WG | TS | CK | JD | LY | FL | Mean±SD | WC | WG | TS | CK | JD | LY | FL | Mean±SD |  |
| **Temperature (°C)*** | **26.91** | **26.79** | **27.22** | **27.63** | **27.93** | **27.85** | **28.39** | **27.53±0.6** | **24.27** | **24.69** | **25.31** | **26.11** | **26.58** | **26.54** | **26.62** | **25.73±1.0*** | ***P* < 0.05** |
| Salinity (psu) | 34.0 | 33.97 | 34.1 | 34.22 | 34.01 | 34.04 | 33.82 | 34.02±0.1 | 33.88 | 34.09 | 34.09 | 34.07 | 33.99 | 34.04 | 34.00 | 34.02±0.1 | NS |
| **pH*** | **8.36** | **8.39** | **8.42** | **8.42** | **8.42** | **8.4** | **8.42** | **8.4±0.01** | **8.2** | **8.17** | **8.2** | **8.18** | **8.22** | **8.19** | **8.18** | **8.19±0.01** | ***P* < 0.05** |
| NO_3_-N (mg l^−1^) | 0.02 | 0.01 | 0.01 | 0.01 | 0.01 | 0.01 | 0.03 | 0.01±0.01 | 0.04 | 0.03 | 0.02 | 0.01 | 0.01 | 0.02 | 0.02 | 0.02±0.01 | NS |
| NO_2_-N (mg l^−1^) | 0.002 | 0.001 | 0.001 | nd | nd | 0.002 | nd | 0.002±0.0006 | 0.010 | 0.010 | 0.002 | 0.001 | nd | 0.002 | 0.002 | 0.003±0.005 | NS |
| PO_4_-P (mg l^−1^) | nd | nd | nd | nd | nd | nd | nd | nd | 0.002 | nd | nd | nd | nd | nd | nd | 0.002 | NS |
| SiO_2_-Si (mg l^−1^) | 0.11 | 0.07 | 0.02 | 0.03 | 0.04 | 0.08 | 0.07 | 0.06±0.01 | 0.13 | 0.11 | 0.08 | 0.05 | 0.04 | 0.06 | 0.06 | 0.08±0.03 | NS |
| **NH_3_-N (mg l^−1^)*** | **0.003** | **0.006** | **0.003** | **nd** | **0.002** | **0.012** | **0.006** | **0.005±0.004** | **nd** | **0.010** | **0.010** | **0.010** | **0.030** | **0.030** | **0.010** | **0.01±0.01** | ***P* < 0.05** |
| Chl-*a* (μg l^−1^) | 0.11 | 0.15 | 0.39 | nd | 0.21 | nd | 0.17 | 0.16±0.10 | 0.10 | 0.23 | 0.17 | 0.32 | 0.77 | 0.4 | 0.18 | 0.31±0.20 | NS |
| **SS (mg l^−1^)*** | **8.39** | **7.94** | **6.78** | **7.39** | **7.33** | **11.72** | **5.56** | **7.87±1.90*** | **11.78** | **24.33** | **30.28** | **13.33** | **9.78** | **12.72** | **9.33** | **15.94±8.10*** | ***P* < 0.05** |
| Clay (%) | 3.15 | 5.79 | 0.61 | 0.88 | 3.45 | 16.47 | 12.07 | 6.06±6.00 | 1.20 | 14.33 | 0.62 | 0.76 | 5.82 | 8.63 | 5.06 | 5.20±5.00 | No test |
| Silt (%) | 15.61 | 25.03 | 4.1 | 5.3 | 17.76 | 74.71 | 61.34 | 29.12±27.80 | 26.41 | 58.98 | 4.17 | 4.55 | 26.52 | 44.08 | 31.85 | 28.08±19.8 | No test |
| Very fine sand (%) | 21.56 | 19.23 | 9.56 | 8.33 | 31.52 | 6.16 | 15.77 | 16.02±8.90 | 21.19 | 9.16 | 16.25 | 10.1 | 36.63 | 26.9 | 39.16 | 22.77±12.0 | No test |
| Fine sand (%) | 15.39 | 41.5 | 48.85 | 42.03 | 31.07 | 2.56 | 10.43 | 27.4±18.0 | 3.31 | 14.74 | 66.19 | 36.63 | 24.61 | 18.65 | 23.51 | 26.81±20.1 | No test |
| Medium sand (%) | 20.87 | 7.33 | 30.33 | 40.17 | 13.24 | 0.09 | 0.4 | 16.06±15.20 | 22.88 | 2.06 | 11.22 | 39.2 | 5.38 | 1.34 | 0.42 | 11.79±14.4 | No test |
| Coarse sand (%) | 21.67 | 1.1 | 6.1 | 3.27 | 2.88 | 0.0 | 0.0 | 5.0±7.7 | 21.79 | 0.73 | 0.52 | 6.2 | 0.97 | 0.38 | 0.0 | 4.37±8.0 | No test |
| Very coarse sand (%) | 1.75 | 0.01 | 0.45 | 0.01 | 0.09 | 0.0 | 0.0 | 0.33±0.6 | 3.23 | 0.0 | 1.03 | 2.56 | 0.06 | 0.0 | 0.0 | 0.98±1.4 | No test |
| Organic matter (%) | 2.02 | 2.27 | 2.16 | 2.21 | 3.06 | 3.89 | 3.76 | 2.77±0.8 | 1.95 | 3.66 | 2.08 | 2.28 | 4.29 | 3.54 | 3.53 | 3.05±0.9 | No test |

Note: The symbol “*“ and bold font indicate significant differences (*p* < 0.05) and NS means *p* > 0.05 in the environmental parameter between the two cruises as tested by paired *t*-test. Abbreviation for site name: WC = Wuchi, WG = Wanggon, TS = Taisi, CK = Chiku, JD = Jiading, LY = Linyuan, FL = Fangliao.

Appendix 3. The correlation matrix (Pearson) and associated *p*-values for the environmental variables used in the canonical correspondence analysis (CCA) in this study.

| Variables | Temperature | Salinity | Chl-*a* | SS | OM | Clay & Silt & Very fine sand | Fine sand | Medium sand & Coarse sand |
| --- | --- | --- | --- | --- | --- | --- | --- | --- |
| Temperature | **1** | −0.162 | −0.099 | **−0.685** | 0.277 | 0.127 | 0.063 | −0.300 |
| Salinity |  | **1** | 0.005 | 0.346 | −0.333 | **−0.632** | **0.589** | 0.424 |
| Chl-*a* |  |  | **1** | −0.009 | 0.387 | 0.007 | 0.277 | 0.058 |
| SS |  |  |  | **1** | −0.059 | −0.056 | 0.158 | −0.020 |
| OM |  |  |  |  | **1** | **0.809** | −0.339 | **−0.800** |
| Clay & Silt & Very fine sand |  |  |  |  |  | **1** | **−0.686** | **−0.848** |
| Fine sand |  |  |  |  |  |  | **1** | 0.448 |
| Medium sand & Coarse sand |  |  |  |  |  |  |  | **1** |
| *p*-values (Pearson) |  |  |  |  |  |  |  |  |
| Variables | Temperature | Salinity | Chl-*a* | SS | OM | Clay & Silt & Very fine sand | Fine sand | Medium sand & Coarse sand |
| Temperature | **0** |  |  |  |  |  |  |  |
| Salinity | 0.580 | **0** |  |  |  |  |  |  |
| Chl-*a* | 0.736 | 0.986 | **0** |  |  |  |  |  |
| SS | **0.007** | 0.225 | 0.975 | **0** |  |  |  |  |
| OM | 0.337 | 0.244 | 0.171 | 0.841 | **0** |  |  |  |
| Clay & Silt & Very fine sand | 0.664 | **0.015** | 0.980 | 0.849 | **0.000** | **0** |  |  |
| Fine sand | 0.831 | **0.027** | 0.338 | 0.590 | 0.236 | **0.007** | **0** |  |
| Medium sand & Coarse sand | 0.297 | 0.130 | 0.843 | 0.947 | **0.001** | **0.000** | 0.108 | **0** |
| Note: Values in bold are different from 0 with a significance level alpha = 0.05. | | | | |  |  |  |  |
